# Clinical Isolates of *Mycobacterium tuberculosis* Differ in Their Ability to Induce Respiratory Burst and Apoptosis in Neutrophils as a Possible Mechanism of Immune Escape

**DOI:** 10.1155/2012/152546

**Published:** 2012-06-21

**Authors:** María M. Romero, Luciana Balboa, Juan I. Basile, Beatriz López, Viviana Ritacco, Silvia S. de la Barrera, María C. Sasiain, Lucía Barrera, Mercedes Alemán

**Affiliations:** ^1^IMEX-CONICET-ANM, Academia Nacional de Medicina, 1425 Buenos Aires, Argentina; ^2^Servicio de Micobacterias, Hospital Malbrán, Pacheco de Melo 3081, 1425 Buenos Aires, Argentina

## Abstract

Tuberculosis pathogenesis was earlier thought to be mainly related to the host but now it appears to be clear that bacterial factors are also involved. Genetic variability of *Mycobacterium tuberculosis* (*Mtb*) could be slight but it may lead to sharp phenotypic differences. We have previously reported that nonopsonized *Mtb* H37Rv induce apoptosis of polymorphonuclear neutrophils (PMNs) by a mechanism that involves the p38 pathway. Here we evaluated the capability to induce PMN apoptosis of two prevalent *Mtb* lineages in Argentina, the Latin America and Mediterranean (LAM), and Haarlem, using the H37Rv as a reference strain. Results showed that LAM strains strongly induced apoptosis of PMN which correlated with the induction of reactive oxygen species (ROS) production and p38 activation. Interestingly, the highly prosperous multidrug-resistant M strain, belonging to the Haarlem lineage, lacked the ability to activate and to induce PMN apoptosis as a consequence of (1) a weak ROS production and (2) the contribution of antiapoptotic mechanisms mediated at least by ERK. Although with less skill, M is able to enter the PMN so that phenotypic differences could lead PMN to be a reservoir allowing some pathogens to prevail and persist over other strains in the community.

## 1. Introduction

Polymorphonuclear neutrophils (PMNs) are key components of the first line of defense against bacterial and fungal pathogens. In tuberculosis (TB), the influx of PMN to the lung is one of the first events in the pathogenesis of the disease. Bacterial products elicit the upregulation of the *β*2 integrin, CD11b, as well as the release of pronflammatory cytokines by PMN contributing to the recruitment of leukocytes at the site of infection and amplifying the immune response [1, 2]. Microbicidal mechanisms of PMNs include the release of proteolytic enzymes and antimicrobial peptides as well as the rapid production of reactive oxygen species (ROS) essential for bacterial killing which also enhance inflammatory reactions [3].

We have previously demonstrated that nonopsonized virulent *Mycobacterium tuberculosis *(*Mtb*) strain H37Rv induces PMN activation and accelerates their apoptosis *in vitro *at a low *Mtb* : PMN ratio [4]. Moreover, in infected individuals circulating PMN become activated [5] and are recruited to the lungs in the early infection, where they underlie apoptosis [6]. Accelerated apoptosis has been observed in PMN after mycobacterial internalization, possibly dependent on oxidative processes [4, 7], enhanced by high concentrations of TNF-alpha [8], and mediated via TLR2- and *mitogen-activated protein kinase* (MAPK) p38- dependent pathways [9]. Along these lines, it has been demonstrated that PMN response to cytokines and other released pro-inflammatory agents as well as activation of PMN involves the MAPK pathway [10]. In the context of mycobacterial infection, the mode of PMN cell death could influence disease outcome: if a PMN cannot kill an ingested organism, then necrosis could offer an inflammatory reaction whereas, if PMN became apoptotic, then PMN-dendritic cell cross-presentation and increased lymphocyte proliferation occurs in response to *Mtb*, directly influencing the development of an acquired immune response [11]. 

In Argentina a total of 11,464 new cases of TB were reported in 2006, with an incidence of 29.1 per 100,000 inhabitants. As in other South American countries [2, 12] the vast majority of *Mtb *strains circulating in Argentina belong to the Latin American and Mediterranean (LAM) and the Haarlem lineages [2, 13, 14]. Within the Haarlem lineage, the multidrug-resistant (MDR) M strain is highly prosperous in Argentina and is able to build up further drug resistance without impairing its ability to spread [15]. The genetic variability of *Mtb* strains might lead to sharp phenotypic differences which could be responsible for a differential modulation of host immune response impacting in the onset and progression of the disease [16]. In this context, differential immune responses among resistant *Mtb* strains have been reported [17, 18]. 

Therefore, in this work we evaluated whether clinical isolates of *Mtb* differ in their capability to induce PMN apoptosis, in order to evaluate apoptosis as a possible mechanism that leads to pathogen survival inside PMN allowing some strains to prevail and persist over other strains in the community. 

## 2. Methods

### 2.1. Isolate Selection and Preparation of Bacterial Strains

**Figure 1:**
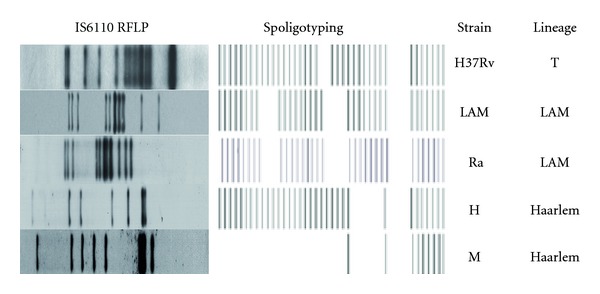
Spoligotyping and IS6110-RFLP pattern profiles of *Mtb* strains used as antigens in this study: reference virulent strain H37Rv, local-drug-sensitive (LAM, 10406) and drug-resistant (Ra, 11608) LAM strains, drug-sensitive Haarlem strain (H, 12425), and MDR Haarlem strain, M (6548).

*Mtb* isolates representative of prevalent LAM and Haarlem lineages were obtained from sputum-culture-positive patients with TB in Argentina. The isolates had been previously submitted to drug susceptibility testing and genotyping by IS*6110 *DNA fingerprinting and spoligotyping using standardized protocols. The following strains were evaluated: a drug-susceptible strain (LAM, 10406) and a drug-resistant strain (Ra, 11608) from the LAM lineage and a drug-susceptible Haarlem strain (H, 12425) and an MDR strain from the Haarlem lineage (isolate 6548 as representative of the outbreak strain M). Spoligotyping and IS*6110* restriction fragment length polymorphism pattern profiles of *Mtb *strains used as antigens are depicted in Figure 1. The strains belonged to the collection kept at the Reference Laboratory for Mycobacteria at the Instituto Nacional de Enfermedades Infecciosas ANLIS “Carlos G. Malbran” in Buenos Aires, Argentina. Laboratory reference strain H37Rv from T family was kindly provided by I. N. de Kantor (Former Head of TB Laboratory, INPPAZ PAHO/WHO) (Figure 1). All strains were grown in Middlebrook 7H9 broth (Difco Laboratories, Detroit, MI, USA) at 37°C in 5% CO_2_ until log phase. Mycobacteria were harvested, washed three times, and suspended in phosphate-buffered saline (PBS) free of pyrogen. Bacteria were killed by gamma irradiation or heat-killed and suspended in PBS at an OD_600_ nm of 1 (~10^8^ bacteria/mL) and stored at −20°C until their use. LPS from *Escherichia coli *0111:B4 was purchased from Sigma Chemicals Co. (St. Louis, USA).

### 2.2. Antibodies and Reagents

Oxidase inhibitor, diphenylene iodonium DPI, was provided by Cayman Chemical (Michigan, USA), the specific inhibitor of p38, SB203580, and the specific inhibitor of ERK, PD98059, were purchased from Calbiochem-Behring (La Jolla, CA, USA). DMSO (Sigma Co.) was added to cultures at 0.1% (vol/vol) as a solvent control. 

Mouse antibodies (Abs) against Caspase-3 (BD Biosciences Pharmingen, California, USA) phospho (Thr202/Tyr204)-ERK1/2, and phosphor (Thr180/Tyr182)-p38 were purchased from Santa Cruz Biotechnology (Santa Cruz, CA, USA). Mouse antihuman CD11b, CD66b and CD16 were purchased from eBioscience (San Diego, CA, USA). 

### 2.3. PMN Purification and Culture

PMNs were isolated from heparinized venous blood from healthy donors by Ficoll-Hypaque gradient centrifugation [19] followed by sedimentation in 3% dextran (Sigma Chem. Co, St. Louis Mo, USA). The PMN-rich supernatant was then collected and residual red blood cells were removed by hypotonic lysis. The cells were washed immediately and resuspended at 3 × 10^6^ cells/mL in RPMI-1640 medium (Gibco, NY, USA) supplemented with 1% heat-inactivated Fetal Calf Serum (FCS) (Gibco) and 50 *μ*g/mL gentamycin (complete media, CM). The viability was consistently >95% as determined by trypan blue dye exclusion. The purity of the final PMN preparation was up to 95% as assessed by morphological examination by staining with Wright-Giemsa and by FACS light scatter patterns. Cultures were performed by incubating 1 mL of a PMN suspension (3 × 10^6^ cell) in Falcon 2063 tubes stimulated with *Mtb* strains, at different *Mtb* : PMN ratios. 

### 2.4. Surface Cell Staining

Cell surface expression of CD11b and FcRIIIb (CD16) in recently isolated or 3 and 18-h-cultured PMN was evaluated by direct immunofluorescence using saturating concentrations of monoclonal mouse antihuman CD11b-PE- and -CD16-FITC-conjugated antibodies. Briefly, 5 × 10^5^ cells were incubated with the antibody for 20 min on ice. Cells were washed, fixed in 500 *μ*L of 1% paraformaldehyde. Using a FACScan (Becton-Dickinson Immunocytometry Systems, San Jose, CA), 10,000 events were collected in linear mode for forward scatter (FSC) and side scatter (SSC), and log amplification for FL-1 and FL-2. Analysis was performed using the CellQuest software (Becton-Dickinson) and isotype-matched controls were used to determine autofluorescence and nonspecific staining. Results were expressed as percentages of positive cells and as mean fluorescence intensity (MFI). 

### 2.5. Intracellular Cell Staining

The activated cytoplasmic protein caspase-3 was measured in PMN by using a Fix and Perm kit (Caltag, Burlingame, CA, USA). Briefly, 3 × 10^6^ PMNs were incubated with different *Mtb *strains at 1 : 2 *Mtb* : PMN ratio for 5 h and thereafter cells were washed and resuspended in 100 *μ*L solution A (fixation) for 15 min at room temperature. After washing with PBS containing 1% Na azide and 5% FCS, cells were resuspended in solution B (permeabilization) and mouse antihuman FITC-conjugated caspase-3 antibody specific for active caspase-3 (BD Pharmingen). After 20-min incubation on ice in the dark, cells were washed, resuspended in isoflow, and analyzed in the same manner as mentioned above.

Phosphorylated form of p38 and ERK cytoplasmic proteins were measured in permeabilized cells as described above. PMNs were incubated with *Mtb* strains at a 1 : 2 *Mtb* : PMN ratio for 1 h. Thereafter, cells were washed with PBS and resuspended in 100 *μ*L solution A (fixation) for 15 min at room temperature. After washing with PBS containing 1% Na azide and 5% FCS, cells were suspended in solution B (permeabilization) and the mouse anti-human p-p38 IgM anti-p-p38-FITC- or anti-human p-ERK-conjugated antibody (Santa Cruz Biotechnology, Santa Cruz, CA., USA). After 20-min incubation on ice in the dark, cells were washed, resuspended in isoflow and acquired in a flow cytometry and analyzed as previously described.

### 2.6. Detection of Apoptosis Using Annexin-FITC by FACS

The percentage of apoptotic PMN was assessed based on the Annexin V-FITC (Sigma) protein-binding assay. Briefly, 5 *μ*L of Annexin V-FITC (10 *μ*g/mL) and 5 *μ*L of propidium iodide (PI) (250 mg/mL) (Sigma) were added to 1.2 × 10^6^ cells in 500 *μ*L of binding buffer and incubated for 15 min at room temperature in the dark, as previously described [4]. Cells were washed, resuspended in binding buffer acquired in a flow cytometry, as previously described. AV^−^/PI^−^ cell population was regarded as alive, AV^+^/PI^−^ was considered as an early apoptotic population, and the AV^+^/PI^+^ population represented late stage apoptotic or necrotic cells. Alternatively, to modify cellular p38 and ERK activity, SB20358 (20 *μ*M) or PD98059 (50 *μ*M) inhibitors was added to 1 mL PMN suspension before culture, at 37°C for 30 min. When indicated, 10 mM H_2_O_2_ were added 15 min after *Mtb* treatment.

### 2.7. Assay for Oxidative Burst Using Dihydrorhodamine 123 (DHR)

Intracellular ROS levels were measured by Dihydrorhodamine assays 123 (DHR). DHR was dissolved in DMSO at a concentration of 20 *μ*g/mL and stored in aliquots at −70°C until use. Briefly, 5 × 10^5^ PMNs were incubated with 100 *μ*l DHR (5 *μ*g/mL) for 15 min at 37°C. Afterward, gamma-irradiated *Mtb *strains (1 × 10^6^ bacilli/mL) were added to the culture at 1 : 2, 5 : 1, 20 : 1, and 50 : 1 *Mtb* : PMN ratios for additional 90 min. When indicated, heated-killed *Mtb* (Ø*Mtb*) were employed.

### 2.8. Phagocytosis Assay

As an indirect measure of phagocytosis ROS production induced by DHR-labeled *Mtb* was performed. Briefly, *Mtb* were incubated with 5 *μ*g/mL DHR for 30 min at 37°C followed by an extensive washing to remove free DHR and suspended in RPMI 1640 plus 10% FCS. Thereafter, 5 × 10^5^ PMNs were incubated with ^DHR^ *Mtb* strains at a 50 : 1 ratio for 90 min at 37°C, washed and centrifuged at 100 g evaluated immediately in a flow cytometer as previously described.

Direct measure of phagocytosis, was performed by the method described by Busetto et al. [20]. Briefly, 3 × 10^6^/mL PMNs were suspended in media 10% FSC and were incubated at 37°C in a shaking water bath with nonopsonized FITC-labeled *Mtb* strains. PMN-*Mtb* mixtures (ratio 10 : 1) were incubated for 2 hr at 37°C. To stop phagocytosis, aliquots of the incubation mixtures were withdrawn into the tubes used for cytometric analysis containing an equal volume of 250 *μ*g/mL Trypan Blue (TB) dissolved in ice-cold 0.1 M citrate buffer, pH 4.0. After 1 min incubation in ice, the samples were analyzed by flow cytometry. Each sample was collected for 30 seconds at the slowest flow rate to minimize the coincidental appearance of free bacteria and PMN in the laser beam. The data were then analyzed by using CellQuest software from Becton Dickinson. The mean number of ingested bacteria per neutrophil was assessed (i.e., both attached and internalized particles).

### 2.9. Statistics

The statistical analysis of the data was performed using one-way ANOVA followed by Tukey's multiple comparison tests to compare more than two groups followed by Wilcoxon test to compare two groups. The significance was considered as *P* < 0.05. The graphical representation of the values is given by mean ± SEM.

## 3. Results

### 3.1. PMN Apoptosis Induced by Different *Mtb* Strains

**Figure 2:**
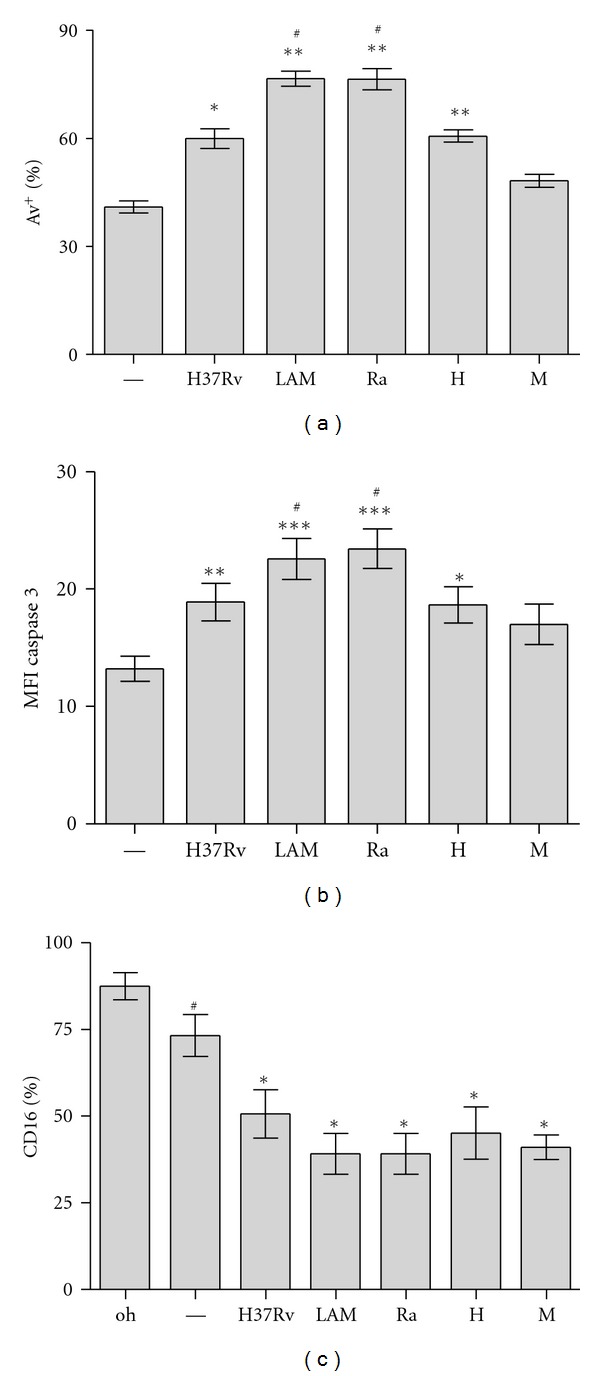
PMN apoptosis induced by different *Mtb *strains. PMNs (3 × 10^6^/mL) were cultured in media alone (control, —) or stimulated with *Mtb *strains (H37Rv, LAM, Ra, H, and M) at 1 : 2 *Mtb* : PMN ratio and parameters of apoptosis were evaluated by flow cytometric analysis. (a) Expression of Annexin V-FITC (AV-FITC) on PMN cultured for 18 h. Results are expressed as media ± SEM of percentage of positive cells (*n* = 20). Control (—) versus LAM, Ra, and H: **P < 0.001; H37Rv: *P < 0.01; LAM and Ra versus other strains: ^#^ *P* < 0.001. (b) Intracellular expression of activated cytoplasmic protein caspase-3 in PMN after 5 h culture with or without *Mtb* stimulation. Results are expressed as media ± SEM of MFI. Control (—) versus LAM and Ra: ***P < 0.001; H37Rv: **P < 0.01; H *P < 0.05; LAM and Ra versus other strains: ^#^ *P* < 0.001. (c) The percentage of fresh (0 h) and 16 h cultured cells expressing FcRIIIb (CD16) was measured. 0 h versus control: ^#^ *P* < 0.0002; control vesrus *Mtb *strains: *P < 0.0001.

We have previously described that nonopsonized strain *Mtb* H37Rv is able to induce PMN apoptosis at low *Mtb* : PMN ratio [4]. In order to evaluate whether *Mtb* strains differ in their capability to induce apoptosis, PMNs were incubated at 1 : 2* Mtb* : PMN ratio for 18 h and, thereafter, apoptosis were evaluated by measuring the percentage of cells expressing FITC-conjugated Annexin V (AV^+^). Flow cytometric analysis shows that all strains, except M, induced significant increase of AV^+^ PMNs respect to spontaneous apoptosis. Furthermore, clinical isolates belonging to LAM lineage (LAM and Ra) were the highest inducers of apoptosis (Figure 2(a)). LPS, that exerts an antiapoptotic effect on cultured PMN [21], was used as a control in the apoptosis assay (data not shown). Most apoptotic signaling pathways are originated from death receptor linkage or stress stimuli converging on activation of caspases, key executors of apoptosis. To assess the involvement of Caspase-3 (casp-3) in *Mtb*-induced PMN apoptosis, expression of the activated form of Casp-3 was evaluated in PMN incubated with different *Mtb* strains (*Mtb* : PMN 1 : 2) for 5 h. As it is shown in Figure 2(b), all strains, except M, induced Casp-3 activation being LAM and Ra the highest inducers. These results are consistent with those obtained in the apoptosis assay. In addition, no differences were observed in the loss of Fc*γ*RIIIb receptor (CD16) expression among strains. As it is known, the loss of CD16 expression on PMN surface correlates with spontaneous apoptosis in culture [22] so that our results show differences in *Mtb*-induced apoptosis whereas CD16 shedding was independent of the *Mtb* strain (Figure 2(c)).

### 3.2. CD11b, CD66b, and p-p38 Expression Induced by *Mtb* Strains

**Figure 3:**
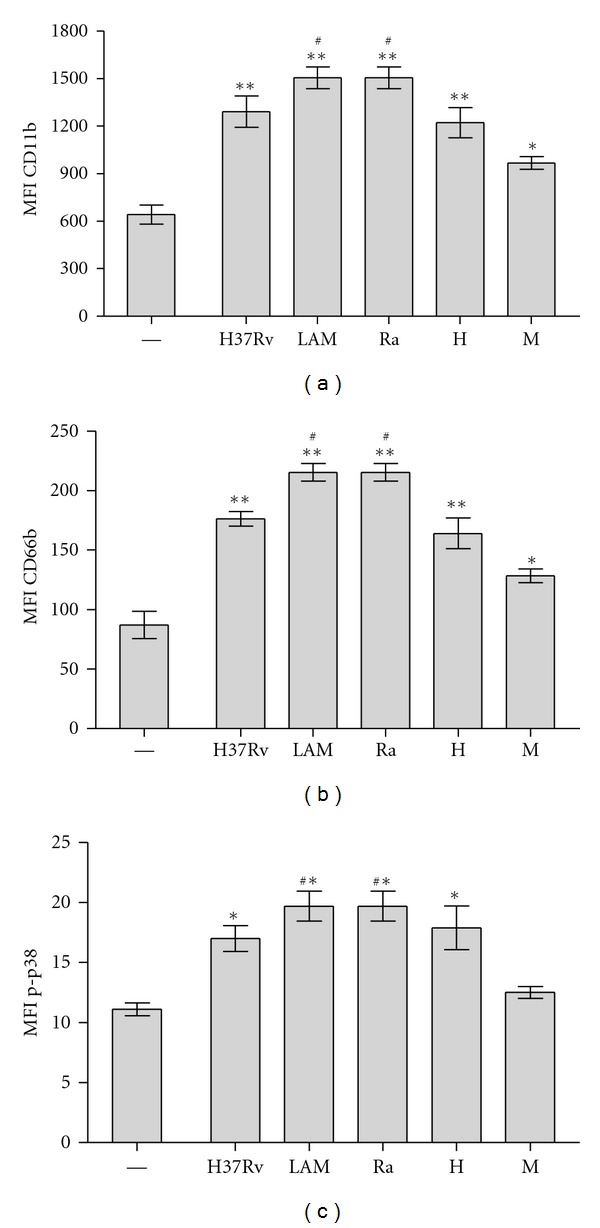
CD11b, CD66b, and p-p38 MAPK expression in PMN induced by *Mtb *strains. PMNs (3 ×10^6^/mL) were cultured in media alone (control, —) or stimulated with *Mtb *strains at 1 : 2 *Mtb* : PMN ratio for 3 h. (a) and (b) Surface expression of activation markers was evaluated by flow cytometry and results are expressed as media ± SEM of MFI (*n* = 10). Statistical differences: CD11b and CD66b: control (—) versus H37Rv, LAM, Ra and H: **P < 0.001; control versus M: *P < 0.05; LAM and Ra versus other strains: ^#^ *P* < 0.01; (c) PMNs (3 × 10^6^/mL) were cultured in media alone (control, —) or stimulated with *Mtb *strains at 1 : 2 *Mtb* : PMN ratio for 1 h. Intracellular activated form of p38 (p-p38) was measured by flow cytometry. Results are expressed as media ± SEM (*n* = 10). Statistical differences: control versus H37Rv, LAM, Ra, and H: *P < 0.001; LAM and Ra versus other strains: ^#^ *P* < 0.001.

CD11b and CD66b are proteins found in the membrane of the various PMN granules and appear on the cell surface after exocytosis and release of granular contents upon activation [23]. Indeed, the CD11b receptor is dramatically upregulated at the surface of activated PMN which extravasate through the vessels and migrate to the site of infection, thereby enhancing the effectors' functions of these cells. As shown in Figure 3, all *Mtb* strains enhanced CD11b and CD66b expression in 3 h culture PMN. Particularly, we found that susceptible and drugs-resistant LAM strains showed the highest activation level, while M showed the lowest one (Figures 3(a) and 3(b)). As previously demonstrated, activation- and apoptosis-induced H37Rv involves the activation of mitogen-activated protein kinase p38 [4, 9]. In this work, flow cytometric analysis of the activated form of p38 (p-p38) showed that susceptible and drugs resistant LAM induced the highest expression of p-p38 whereas, with M, p-p38 was not detectable. These results suggest a different participation of p38 in apoptosis induced by *Mtb* strains (Figure 3(c)). From now on, we will refer to both LAM and Ra as “LAM,” because no differences were found between susceptible or drugs-resistant LAM clinical isolates.

### 3.3. Role of p-p38, ERK, and ROS in Differential* Mtb*-Induced PMN Apoptosis

**Figure 4:**
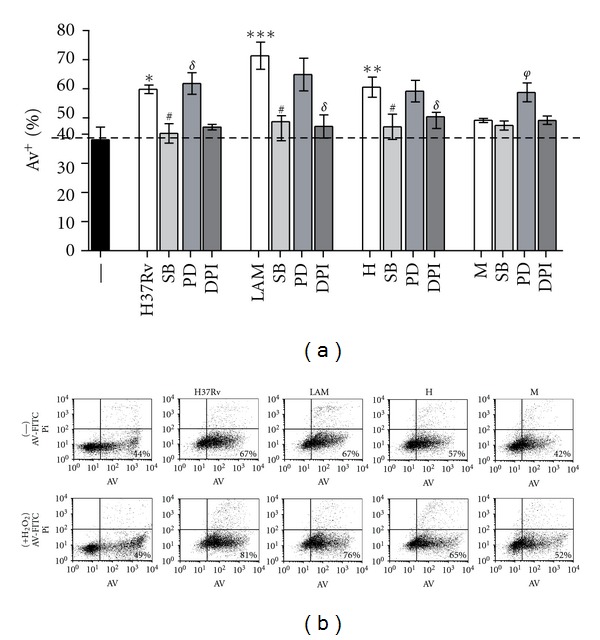
Role of MAP kinases and reactive oxygen species in PMN apoptosis induced by *Mtb *strains. (a) PMN (3 × 10^6^/mL) were cultured in media alone (control, —) or stimulated with *Mtb *strains at 1 : 2 *Mtb* : PMN ratio for 18 h, in the presence or absence of specific inhibitors for p38 and ERK (SB203580 and PD98059, resp.), and an oxidase inhibitor DPI. Thereafter, Annexin V-FITC (AV-FITC) binding was measured by flow cytometry. Results are expressed as media ± SEM (*n* = 12). Statistical differences: Control versus LAM: ***P < 0.001, control versus H: **P < 0.01, control versus H37Rv: *P < 0.05; H37Rv, LAM and H versus SB: ^#^ *P* < 0.01; H37RV, LAM and H versus DPI: ^*σ*^ *P* < 0.001; M versus PD: ^*φ*^ *P* < 0.01. (b) PMN were treated with 10 mM H_2_O_2_ 15 min before *Mtb* challenge and thereafter PMN were cultured as described in (a). After 18 h-culture, Annexin V-FITC (AV-FITC) positive PMN was measured by flow cytometry. A representative experiment out of four is displayed.

To evaluate if *Mtb* strains differ in the mechanisms underlying the apoptotic process, PMNs were cultured with *Mtb* in presence of different inhibitors for 18 h and apoptosis was evaluated by AV expression. According to our previous results obtained with H37Rv [9], SB203580, a specific p38 inhibitor, only inhibited the apoptosis induced by LAM and H (Figure 4(a)). Considering that ROS are involved in the regulation of MAP-kinase-dependent apoptotic pathway [24], we used an oxidase inhibitor (DPI) to assess the participation of ROS in apoptosis and we observed that generation of ROS was essential for triggering apoptosis induced by all strains (Figure 4(a)). Strikingly, M was able to induce apoptosis when PMNs were incubated with a specific ERK inhibitor, PD98059, suggesting that this strain could be triggering anti-apoptotic mechanisms, which involve ERK (Figure 4(a)). In order to evaluate the grade of participation of anti-apoptotic signals, H_2_O_2_ was added to the culture before challenging with different *Mtb* strains and apoptosis was measured by AV. Interestingly, apoptosis induced by all strains was enhanced in the presence of H_2_O_2_. However this effect was lower for the Haarlem strains, in particular M, which effect was not enough to reach high levels of apoptosis (Figure 4(b)), supporting the idea that anti-apoptotic mechanisms could be involved.

### 3.4. Role of p-p38, ERK, and ROS in CD11b UpRegulation by* Mtb* Strains

Previously we have shown that H37Rv-induced apoptosis depends on PMN activation as *Mtb* induced also CD11b expression [4]. CD11b is a receptor that serves in nonopsonic recognition of microbes which may confer an advantage in the alveolar space, where serum opsonins are limited. We further evaluated whether the activation of p38 is involved in the upregulation of CD11b expression and, as observed in Table 1, SB203580 abolished CD11b upregulation by all strains. Besides, PD inhibited M-induced C11b enhancement suggesting that ERK was also required for M. This data is in accordance with that observed to apoptosis. However, CD11b expression was not abrogated by DPI suggesting that ROS did not participate in CD11b upregulation. 

### 3.5. ROS Production Induced by *Mtb* Strains in PMN

**Figure 5:**
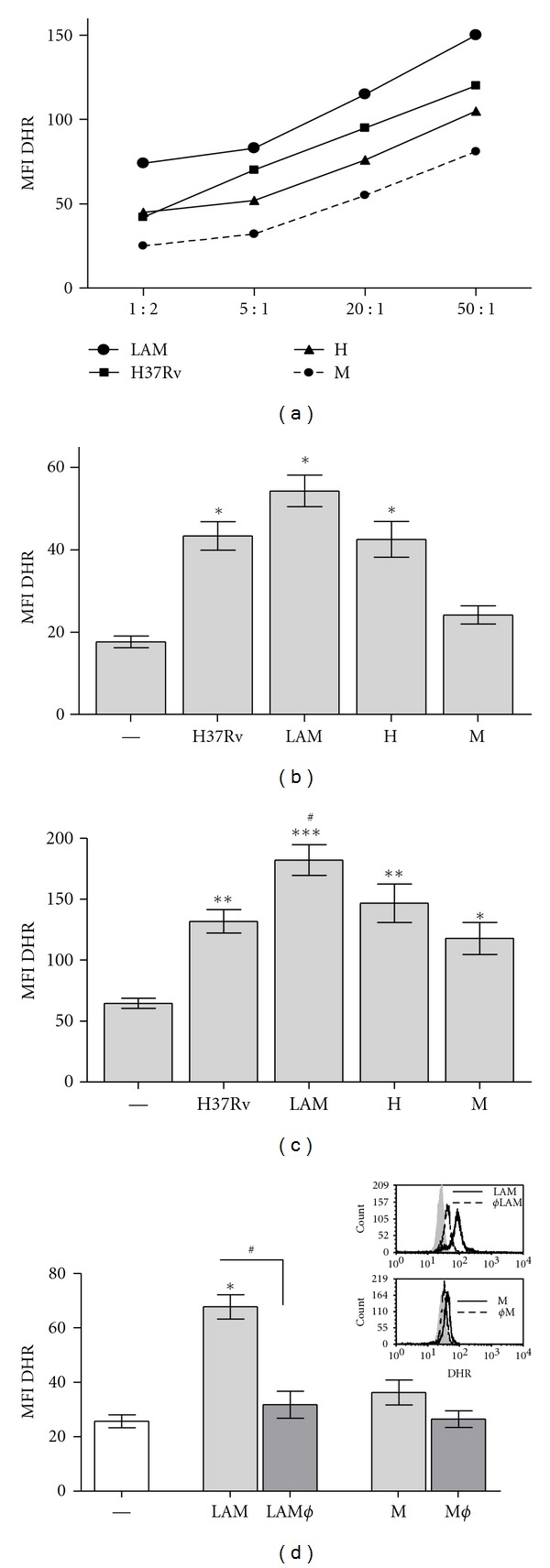
ROS production induced by *Mtb *strains in PMN. (a) PMNs treated with DHR were incubated with *Mtb *strains and MFI of 123rodhamine was measured by flow cytometry. Different *Mtb* : PMN ratios were tested (1 : 2, 5 : 1, 20 : 1, and 50 : 1) and a representative experiment out of three is shown. (b) ROS production induced at low *Mtb* : PMN ratio (1 : 2); results are expressed as media ± SEM (*n* = 20), statistical differences: control versus LAM, H37Rv, and H: *P < 0.001. (c) ROS production induced at 50 : 1 *Mtb* : PMN ratio; results are expressed as media ± SEM (*n* = 20), statistical differences: control versus LAM ***P < 0.001; H37Rv and H **P < 0.01; M *P < 0.05. (d) ROS production was induced with gamma-irradiated or heat-killed (Ø) *Mtb *strains at 1 : 2 *Mtb* : PMN ratio. Statistical differences: control (—) versus LAM *P < 0.001; LAM versus LAMØ ^#^ *P* < 0.01. A representative experiment is embedded. In all cases PMNs were washed, and 10,000 events were collected in a flow cytometer as described in Section 2.

Considering that apoptosis in human PMN may be related to ROS production [25] we wondered if M, that fails in induce PMN apoptosis, would exhibit any deficiency to induce ROS. Therefore, we evaluated the conversion of nonfluorescent dihydrorhodamine123 (DHR) to rhodamine123 as a measure of H_2_O_2_ production. DHR freely enters the cell; it binds to cellular and mitochondrial membranes and emits a bright fluorescent signal mainly localized inside the cell [26]. As it is shown in Figure 5(a), all *Mtb* strains induced ROS production in a dose-dependent manner. At low *Mtb* : PMN ratio (1 : 2), LAM, H, and H37Rv strains were able to induce significant amount of ROS whereas M did not (Figure 5(b)). Besides, at high doses (50 : 1 *Mtb* : PMN) all *Mtb* strains were able to induce ROS, being LAM the highest inducer (Figure 5(c)).

### 3.6. Phagocytosis of *Mtb* Strains by PMN

On the other hand, it is known that certain pathogens fail to trigger ROS and apoptosis in PMN and enter the cell at a low rate [27], so that we wondered whether *Mtb* strains exhibited a disparity in their entrance to the PMN. In this purpose, PMNs were incubated with FITC-labeled *Mtb* during two hours and the percentage of PMN expressing FITC-*Mtb *was evaluated by flow cytometry. As shown in Figure 5(d), M was less engulfed than LAM, H37Rv, and H. Similar results were obtainedinPMNinfected withlive bacteria (data not shown).

**Figure 6:**
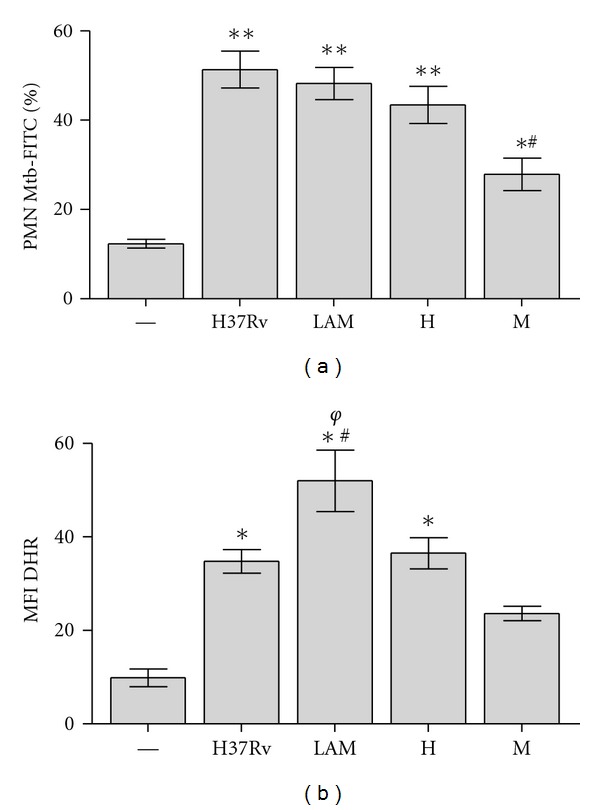
Phagocytosis of *Mtb *strains by PMN (a) As direct measure of phagocytosis, PMN were incubated with FITC-labeled *Mtb* (ratio 10 : 1). Then all *Mtb*-FITC bound/not ingested by PMN were quenched with Trypan Blue. Results represents bound/ingested plus ingested and are expressed as media ± SEM of MFI (*n* = 10). Statistical differences: Control (—) versus H37Rv, LAM and H: **P < 0.001 control versus M *P < 0.05; M versus other strains ^#^ *P* < 0.001. (b) Phagocytosis was indirectly measured by ROS production induced with DHR labeled *Mtb *(DHR*Mtb*). PMN were incubated with DHR*Mtb *(50 : 1 ratio) for 90 min. at 37°C and 123rhodhamine emission was evaluated immediatly in a flow cytometer. Results are expressed as media ± SEM of MFI (*n* = 10). Statistical differences: control (–) versus H37Rv, LAM and H: *P < 0.001; LAM versus H37Rv and H ^#^ *P* < 0.05; LAM versus M ^*φ*^ *P* < 0.001. In all cases PMN were washed, and 10,000 events were collected in a flow cytometer as described in Section 2.

Besides, in order to determine if low ROS production was associated with a low entrance, we quantified ROS with DHR-labeled *Mtb* (^DHR^ *Mtb*), so that oxidized DHR represents the levels of ingested bacteria.  Figure 5(e) shows that while M was not able to induce ROS, LAM was the highest inducer. Although H and H37Rv showed a similar phagocytosis rate as LAM, these two strains induced less ROS production, suggesting that the differences seem not to be related to their efficiency to enter in PMN. In particular, the fact that M was not able to induce ROS in this condition might be related to the low phagocytic rate and a poor ability to induce ROS (Figure 6). 

## 4. Discussion

The outcome of tuberculosis infection has long been considered dependant mainly on the ability of the host to deal with the pathogen. Now it is becoming clear that in addition to host factors, phenotypic differences among *Mtb* strains are also involved in key aspects of pathogenesis and latency as transmissibility, virulence, and immunogenicity among *Mtb *strains, determined by the genetic background of the organisms [15, 28–30]. In this context, the *Mtb* envelope contains diverse lipids and glycolipids that are likely to mediate specific host interactions [31].

PMNs are the first defensive cells recruited into infected tissue, where their role has been thought to be the elimination of invading pathogens via mechanisms such as the generation of ROS [32] and the release of preformed oxidants and proteolytic enzymes from granules [33]. In this way, PMN apoptosis induced by bacteria is likely beneficial to the host because it reduces the release of cytotoxic components [34] and promotes PMN removal by macrophages from sites of infection, reducing tissue destruction during necrosis. Microorganisms that evade killing survive and disseminate to cause disease [35].

The aim of this work was to evaluate the prevalent *Mtb* strains in Argentina in their capability to induce PMN apoptosis compared with H37Rv. As we showed here, except M, all *Mtb* strains induced a significant increase in PMN apoptosis at *Mtb* : PMN ratio (1 : 2). The very high level of apoptosis induced by LAM, both sensible (LAM) and multidrugs-resistant (Ra) strains, was in accordance with a marked activated phenotype of PMN, as well as Casp-3 and p-p38 expression, and moreover, *Mtb*-induced apoptosis has correlated with ROS production. In the same way, M, that fails to induce PMN apoptosis, also fails at inducing expression of Casp-3 and p-p38 and ROS production.

Today it is known that ROS can act as signaling molecules [36] modulating diverse receptor-ligand intracellular signaling pathways, such as the MAPK-dependent apoptotic pathway and NF-kB pathways [2, 24]. In this context it has been described that PPD induces the rapid activation of MAPKs, ROS, and apoptosis signal-regulating kinase 1 (ASK1) in monocytes in a TLR2-dependent manner [37] and ASK1 could also be activated by oxidant stress, involving p38 pathway. A strong evidence for the involvement of ROS in PMN apoptosis is the fact that phagocytes from patients with chronic granulomatous disease that are unable to produce ROS due to a defect in NADPH oxidase are predisposed to recurring bacterial and fungal infections and develop granulomas linked to altered PMN apoptosis [38]. Patients with chronic granulomatous disease are also susceptible to TB and complications of vaccination with the bacillus Calmette-Guérin [39]. 

Here we show that both ROS and p38 are involved in the apoptosis triggered at low *Mtb* : PMN rate. Remarkably, M was able to trigger apoptosis when ERK was abrogated suggesting that M activates anti-apoptotic mechanisms which could also impact on final apoptosis outcome in PMN. This result was supported by the fact that addition of H_2_O_2_ allowed a partial induction of apoptosis by M. The involvement of ERK in signaling triggered by M was also evident as both p38 and ERK were involved in CD11b expression induced by M, whereas other strains only activated p-p38 (Table 1). Although p-p38 induced by M was not detectable, it could be enough to activate PMN but not to overcome antiapoptotic mechanisms triggered by this strain.

It has been described that PMN uptake of the obligated intracellular pathogen *Anaplasma phagocytophilum *occurs at a slow rate compared with other bacteria [27], and even more, PMN infection with this pathogen fails to trigger ROS and delays PMN spontaneous apoptosis [2, 25]. In this context, albeit M was ingested at a lower rate, no differences were observed among LAM, H37Rv, and H (Figure 5(d)), suggesting that differences in ROS production were not related to the efficiency in bacterial entry. Consistently, albeit M was able to induced ROS at high *Mtb* : PMN ratio, it was not able to induce ROS when DHR came from *Mtb* that has been ingested (*Mtb*PMN), because of the sum of the low phagocytic rate and a poor ability to induce ROS. Therefore, we can assume that LAM is more efficient in entering PMN and also has a major capability to induce ROS than other strains. Moreover, it is possible that *Mtb* strains would not be qualitatively different in their capacity to induce ROS but merely show a quantitatively different dose-response curve that is finally reflected in apoptosis outcome.

Pathogens have developed strategies to counteract NADPH oxidase function and suppress generation of phagosomal ROS. The *nuo* G gene encodes the NuoG subunit of the type I NADH (nicotinamide adenine dinucleotide)-dehydrogenase of *Mtb*. Deletion of this gene leads to elevated ROS levels and apoptosis following infection of primary macrophages [40].Similarly, deletion of the *sec*A2 gene, which encodes a protein required for the secretion of superoxide dismutase A, results in enhanced ROS production and apoptosis.Superoxide dismutase A breaks down superoxide leading to diminished ROS generation, which is a mechanism that *Mtb* uses to prevent apoptosis [41]. Nevertheless, our data obtained with heat-killed bacteria suggest that the mechanism exerted by M might not involve those enzymes, but structural differences between clinical isolates leads to different ROS/apoptosis rate. 

LAM and Haarlem are the two main families circulating in Latin American countries [2, 13, 14]. Among them, two clinical isolates differently interact with PMN in the host. In particular, M strain of the Haarlem family can be regarded as a highly successful genotype in Argentina because it has been able to prevail and persist over other MDR *Mtb* strains in the community [42, 43] being able to build up further drug resistance without impairing its ability to spread [15]. In this context, it has been described that, when killing is absent, not only could the disease progress locally, but the infected PMN could also traffic organisms to distal sites, in particular if bacilli reach the systemic circulation. Indeed, a granulocytic “Trojan horse” has been proposed by several authors in the context of mycobacterial infection [44]. Therefore, though less skillfully, M is able to enter the PMN and in turn generate fewer ROS. As a result, PMNs have less capacity to kill M and greater capacity to prolong its life, becoming a reservoir for this strain or another strain that uses this mechanism. 

## 5. Conclusion

In conclusion, here we show that, independently of the ability to entering PMN, certain *Mtb* strains (as LAM) induce high PMN apoptosis by triggering signaling mechanisms that involves ROS generation via p38 activation, leading to enhanced effectors' functions whereas others fail in activating PMN to kill pathogen as well as inducing apoptosis as a consequence of no. (1) a slight ROS production and (2) contribution of anti-apoptotic mechanism mediated at least by ERK, making PMN a “Trojan horse,” which could be a beneficial mechanism allowing some strains to prevail and persist over other strains in the community. 

## Figures and Tables

**Table 1:** CD11b expression on 3 h-cultured PMN stimulated with *Mtb* strains and treated or not with MAPK or ROS inhibitors.

Treatment^a^/ *Mtb* strain	—	H37Rv	LAM	H	M
—	729 ± 107	1300 ± 137*	1529 ± 115*	1234 ± 115^∗#^	992 ± 95^∗#f^
+SB	720 ± 100	805 ± 126	950 ± 120	764 ± 87	574 ± 85
+PD	730 ± 110	1303 ± 176*	1480 ± 160*	1148 ± 132*	722 ± 62
+DPI	770 ± 105	1334 ± 328*	1442 ± 200*	1363 ± 262*	963 ± 120*

^a^PMN (3 × 10^6^/mL) were cultured in media alone (—) or stimulated with *Mtb* strains (H37Rv, LAM, H, M) at 1 : 2 *Mtb* : PMN ratio for 3 h, in the presence or absence of the specific inhibitors of p38 (SB), ERK (PD) or NADPH oxidase (DPI). Thereafter, CD11b expression (MFI) was measured by flow cytometry.

^∗^Statistically significant: (*P* < 0.02) compared with medium alone.

^#^Statistically significant: (*P* < 0.005) compared with H37Rv and LAM.

^f^Statistically significant: (*P* < 0.03) compared with H.

## Abbreviations

**Mtb **: *Mycobacterium tuberculosis*
PMN: polymorphonuclear neutrophils
ROS: Reactive Oxygen Species
TB: tuberculosis
DHR: dihydrorhodamine
MAPK: mitogen-activated protein kinase.
